# Cell surface galectin-3 defines a subset of chemoresistant gastrointestinal tumor-initiating cancer cells with heightened stem cell characteristics

**DOI:** 10.1038/cddis.2016.239

**Published:** 2016-08-11

**Authors:** Matthias Ilmer, Nachman Mazurek, James C Byrd, Karen Ramirez, Margarete Hafley, Eckhard Alt, Jody Vykoukal, Robert S Bresalier

**Affiliations:** 1 Department of Translational Molecular Pathology, University of Texas MD Anderson Cancer Center, Houston, TX USA; 2 Department of Gastroenterology, Hepatology and Nutrition, University of Texas MD Anderson Cancer Center, Houston, TX USA; 3 Department of Stem Cell Transplantation and Cellular Therapies, University of Texas MD Anderson Cancer Center, Houston, TX USA; 4 Department of Medicine, Tulane University Health Science Center, New Orleans, LA USA

## Abstract

Recurrence of gastrointestinal adenocarcinomas after surgery and chemotherapy may be attributed, in part, to the presence of a small population of tumor-initiating cancer stem cells (CSC). The expression of galectin-3 (Gal3), a multifunctional oncolectin, has been associated with biological behaviors associated with CSC. We examined the ability of Gal3 to characterize the CSC phenotype, and to identify a clinically important gastrointestinal cancer CSC population. Human colorectal and pancreatic cancer cell lines were sorted to identify subpopulations expressing commonly used CSC markers, and Gal3-positive CSC subpopulations. The association of Gal3 with the stem cell properties and alterations of these phenotypes by manipulation of Gal3 expression was examined. Gastrointestinal cancer cell lines contain both Gal3-positive and Gal3-negative subpopulations. Gal3-positive CSCs are characterized by high ALDH activity, enhanced self-renewal ability *in vitro* (sphere formation) and tumor forming ability *in vivo*, and resistance to chemotherapeutic agents and death-receptor-mediated apoptosis compared to Gal3-negative CSCs. Silencing Gal3 modifies this behavior. Cell surface Gal3 expression identifies a subset of CSCs in gastrointestinal cancers with high levels of stem cell characteristics, including chemoresistance. This may provide a platform for developing treatment strategies that target CSC.

The clinical management of cancer patients has improved overall survival, but some patients nevertheless become resistant to treatment and develop metastatic disease Recurrence of metastatic colorectal and pancreatic ductal adenocarcinomas after surgery and chemotherapy may be attributed, in part, to the presence of a small population of tumor initiating or cancer stem cells (CSC). These cells are rare and highly heterogeneous in nature and characterized by unlimited self-renewal capability, drug-resistance, as well as potential for tumor progression and metastasis. The definitive identification of clinically relevant CSC subgroups that ultimately reduce patient survival however remains a challenge.

Cancer stem cells have been recognized in liquid and solid tumors as the drivers of tumor growth and drug-resistance with subsequent tumor relapse, and metastasis.^1, 2, 3^ The identification and isolation of CSC, however, remains challenging. Many reputed stem cell markers were empirically determined, may be restricted to certain models, and controversy exists regarding which markers accurately define CSC.^4, 5^

Galectins are carbohydrate-binding proteins characterized by their binding affinity for beta-galactosides and by conserved sequences in the carbohydrate-binding site.^6^ It has been shown that galectin-3 (Gal3) is responsible for a myriad of biological processes in cancer. In colon cancers, Gal3 is distributed in the cytoplasm and nucleus, and is present at the cell surface,^7^ but its specific role in each compartment remains to be determined. Furthermore, tumor and inflammatory cells are able to secrete Gal3. Studies in colorectal cancer have linked increased serum Gal3 with metastatic disease.^8^ In pancreatic cancer, it was shown that Gal3 expression positively correlates with proliferative and invasive behavior.^9^ Recently, a novel resistance mechanism to tumor necrosis factor-related apoptosis-inducing ligand (TRAIL) was reported in which Gal3 blocks the critical trafficking of death receptor signals prior to apoptosis induction rendering high Gal3-expressing cells resistant to TRAIL.^10^ Gal3 appears therefore to functionally and structurally contribute to a number of CSC hallmarks which prompted us to investigate its role in gastrointestinal cancer ‘stemness'.

We report that Gal3 identifies a subset of CSC in gastrointestinal cancers with heightened stem cell characteristics such as sphere-forming ability (SFA), ALDH activity, multiple drug-resistance, and tumorigenesis. This work identifies CSC subsets in solid gastrointestinal tumors, and might help to develop future successful therapeutic CSC-based approaches.

## Results

### Cellular models

For the current work, we chose cell systems representative of solid colorectal and pancreatic epithelial neoplasms. The human colorectal cancer cell line LS-LiM6 (LiM6) was originally established by serial *in vivo* passaging of LS-174T cells that were implanted into the cecum of athymic mice and re-isolated from their liver lesions.^11^ As another representative colorectal cancer cell line, we used the well-described cell line DLD1.^12^ We recently described derivatives of LiM6 and DLD1 that were highly resistant to TRAIL treatments; these sub clones were utilized due to their heightened surface Gal3 expression and named LiM6-TR and DLD1-TR, respectively.^10^

Parallel to the colorectal cancer model, we used a set of pancreatic cancer cells in order to address the question whether our findings are of general relevance in solid gastrointestinal tumors. In a manner similar to the generation of LiM6, L3.6pl was established by serial artificial metastatic enrichment,^13^ whereas AsPC1 was originally developed from an ascites sample from a pancreatic cancer patient.^14^

### Surface Gal3 defines a subset of epithelial stem cells

Cancer stem cells have reportedly been enriched by cell sorting of a small and highly tumorigenic subset of cancer cells using the specific expression of surface markers. In colorectal cancer and pancreatic cancer, CD24, CD44, EpCAM and CD166 are widely used to define stem cells.^15, 16^ Cells with positive expression of all four of these surface markers were therefore termed CSC in our study.

**Figure 1:**
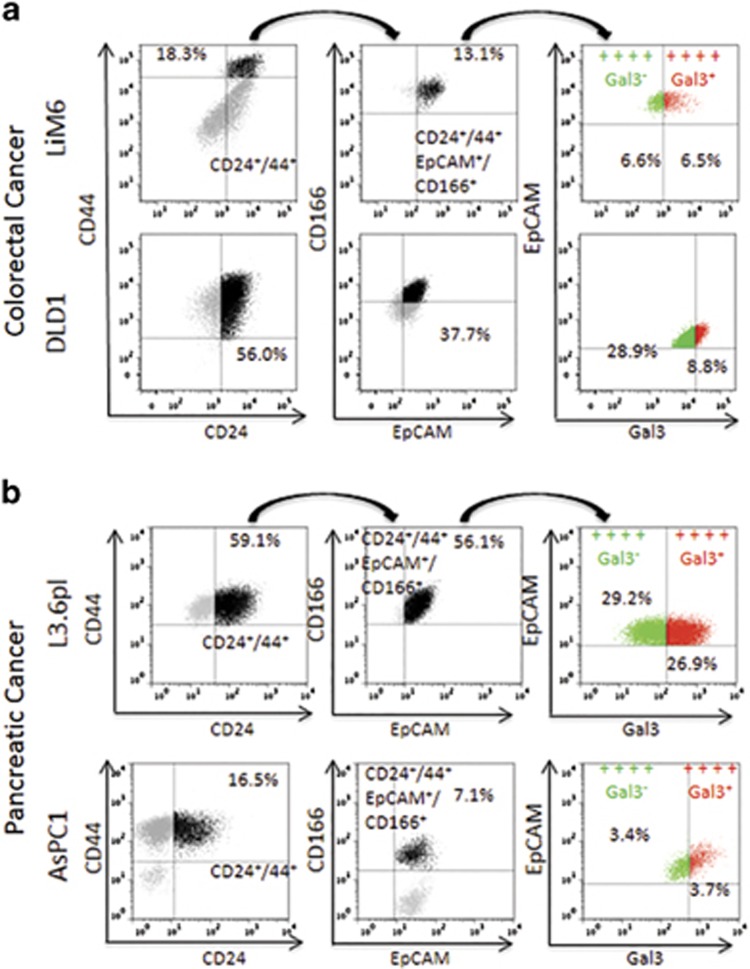
Surface Gal3 defines a subtype of epithelial CSC. (**a**) Colorectal cancer cells LiM6 and DLD1 were investigated by flow cytometry for cell surface markers CD24/CD44 (left). CD24+/CD44+-cells (in black) were then investigated for CD166/EpCAM-expression (middle) and CD24+/CD44+/CD166+/EpCAM+-positive cells (in black middle) for Gal3-expression (right panel), where ++++ Gal3^−^ in green refers to cells positive for CD24, CD44, CD166, and EpCAM, but negative for Gal3, and ++++ Gal3^+^ in red refers to cells positive for CD24, CD44, CD166, EpCAM, and Gal3. (**b**) Pancreatic cancer cell lines AsPC1 and L3.6pl were subjected to the same analysis as in (**a**)

We observed that LiM6 contains a small CD24+/CD44+population (18.3%) of which 71.6% were also positive for CD166 and EpCAM. Therefore 13.1% of the original LiM6 population were considered CSC (Figure 1a upper panel). In DLD1, 56.0% were CD24+/CD44+, 67.3% were in addition CD166^+^/EpCAM^+^, yielding 37.7% CSC (Figure 1a lower panel) among the original DLD1 cells. Within the two CSC groups, we detected Gal3^positive^ (indicated in red) and Gal3^negative^ (indicated in green) subsets. 49.6% of LiM6-derived CSC were Gal3^positive^, while 23.3% of DLD1derived CSC were Gal3^positive^ (Figure 1a, right).

In the pancreatic cancer cells L3.6pl contained 59.1% CD24+/CD44+ cells of which 94.9% were EpCAM+/CD166+, yielding a 56.1% CSC subset (Figure 1b, upper panel). In comparison, we observed only 16.5% CD24+/CD44+-cells in AsPC1 of which 43.0% were CD166+/EpCAM+ yielding a 7.1% CSC-subset (Figure 1b, lower panel). Similar to colon cancer cells, pancreatic CSC contained subsets that were either Gal3^positive^ (indicated in red) or Gal3^negative^ (approximately 50%).

In follow-up experiments we selected CSC by sorting (Supplementary Fig. S1A, Step 1), and then divided these cells into Gal3^positive^ and Gal3^negative^ groups (Step 2). Cell fractions were expanded as spheres, sorted for ALDH^positive^ cells (Step 3), and subsequently propagated as spheres (Step 4). Further analyses were carried out with these Gal3^positive^ CSC or Gal3^negative^ CSC (Step 5). Spheres from Step 4 were periodically checked for their CSC marker and Gal3 expression by Western analysis (protein expression) and flow cytometry (cell surface expression) as well as ALDH activity, and remained stable up to seven generations.

### Gal3^positive^ CSC display increased functional stemness characteristics

**Figure 2:**
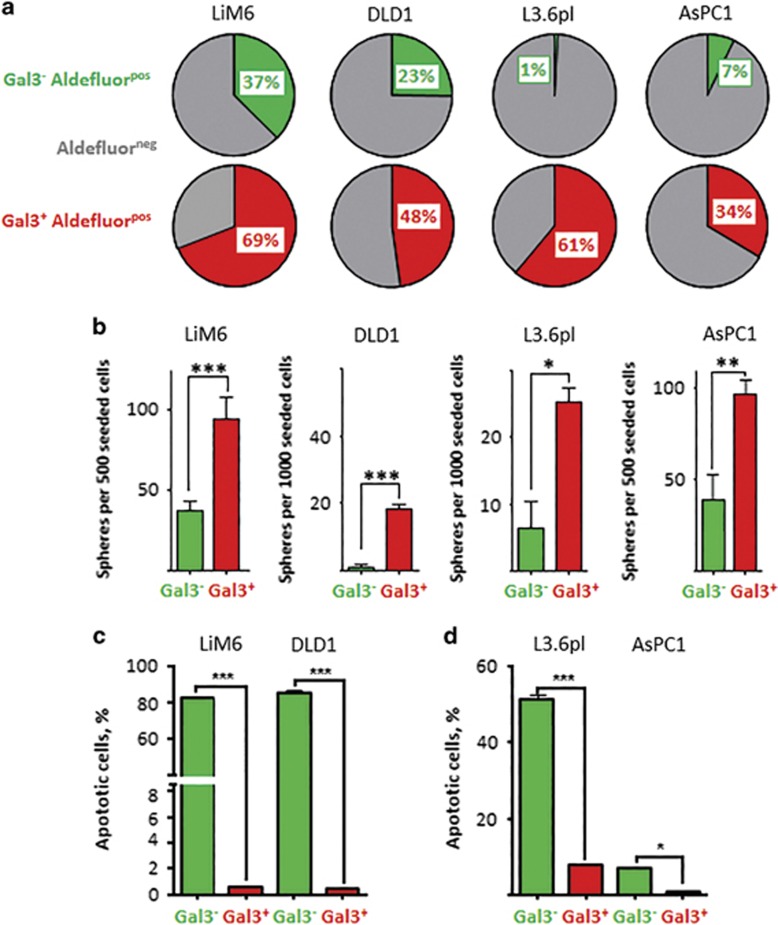
Gal3^positive^ CSC subset displays increased stemness characteristics. (**a**) ALDH activity was evaluated by flow cytometric analysis in Gal3^positive^ CSC (in red) and Gal3^negative^ CSC (in green). ALDH^negative^ fractions are illustrated in gray. (**b**) Sphere formation ability (SFA) was determined. Gal3^positive^ CSC (red) accounted for significantly more spheres than Gal3^negative^ CSC (green). (**c**) TRAIL-resistance in Gal3^positive^/Gal3^negative^ colon cancer CSC subsets was determined by APO-BRDU assay. Apoptotic cells are displayed as percentage of total cell count. (**d**) TRAIL-resistance in Gal3^positive^/Gal3^negative^ CSC pancreatic cancer subsets. All experiments were performed in triplicate (see text for details). *P*<0.05 (*), *P*<0.01 (**), and *P*<0.001 (***)

We next sought to investigate the effect of Gal3 positivity on cancer stemness behavior comparing Gal3^positive^ and Gal3^negative^ CSC *in vitro* using a variety of functional assays. First, using flow cytometry analysis, we analyzed ALDH activity using the ALDEFLUOR assay. ALDH^positive^ cells presumably contain CSC.^17^ In all cell lines, we found that Gal3^negative^ subsets had significantly less ALDH^positive^ cells than their Gal3^positive^ counterparts (LIM6 37.4±2.1% *versus* 69.2±4.6% DLD1 23.6±1.2% *versus* 48.3±2.6% L3.6pl 1.2±0.6% *versus* 61.4±3.7% ASPC1 7.2±0.8% *versus* 34.3±2.4%) (Figure 2a). Another *in vitro* hallmark of stemness is SFA that can be evaluated in an assay that also correlates with anoikis-resistance.^18^ Here, we found that Gal3^positive^ CSC possessed a significantly higher SFA than Gal3^negative^ CSC in all cell lines (Figure 2b).

Next, we sought to investigate drug-resistance to TRAIL, a characteristic that was previously shown to be associated with Gal3 expression,^10^ and is another accepted CSC hallmark.^19^ Gal3 expression led to highly significant differences in TRAIL sensitivity, even within CSC pools. In colorectal CSC, TRAIL treatment led to more than 80% apoptotic cells when these cells were Gal3^negative^ (Figure 2c, in green), while Gal3^positive^ CSC (in red) were virtually unaffected. We observed similar trends in pancreatic cancer cells, but with higher intrinsic TRAIL-resistance particularly for AsPC1 (Figure 2d).

### Elevated levels of Gal3 correlate with high SFA

**Figure 3:**
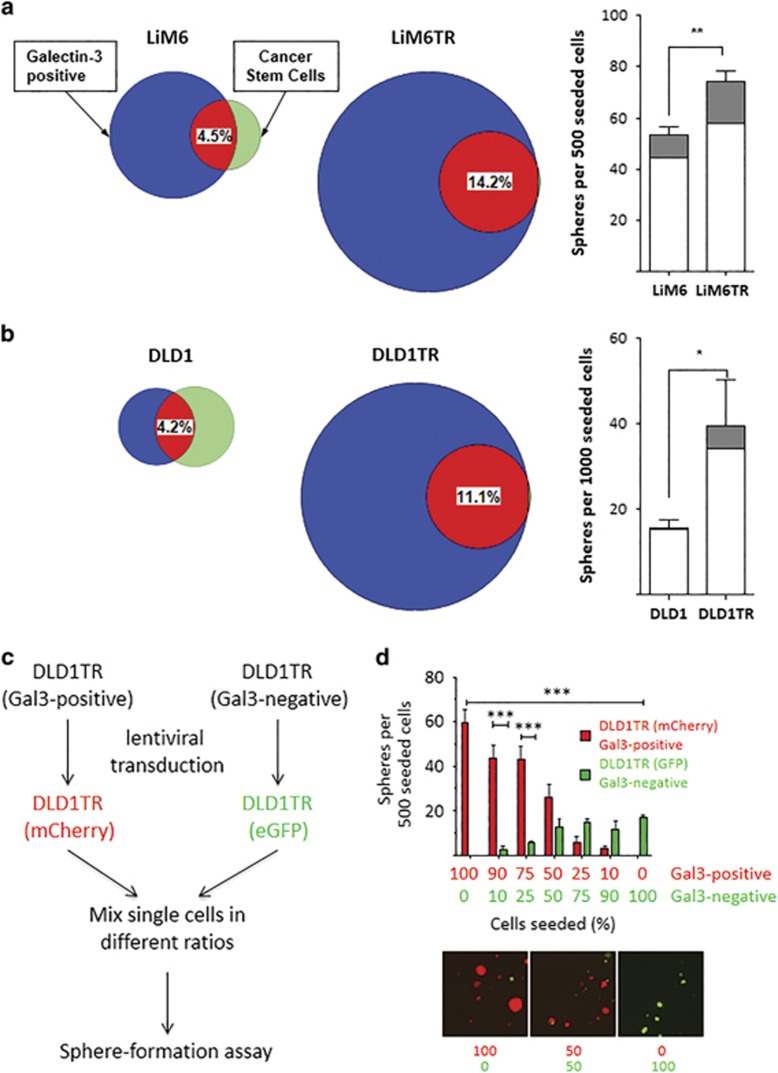
Elevated levels of Gal3 correlate with higher number of CSC marker positive cells. (**a**) *Left:* Venn diagrams, where areas represent percentage of total cell population, display surface Gal3-positive (blue plus red), CSC (green plus red), and their intersection (red) in LiM6 or LiM6TR. *Right:* SFA was determined in parental LiM6 and LiM6TR cells. White bars, spheres of 200–300 *μ*m; black bars, spheres of >300 *μ*m. (**b**) *Left:* Venn diagrams of expression of Gal3 and CSC markers in DLD1 and DLD1TR. *Right:* SFA was determined in parental DLD1 and DLD1TR cells. White bars, spheres of 75-150 *μ*m; gray bars, spheres of >150 *μ*m. (**c**) Lineage-tracking experiment outline: Gal3^positive^ CSC was transduced with an mCherry-lentiviral vector, Gal3^negative^ CSC with an eGFP-lentiviral vector and single cells of both mixed at different ratios. (**d**) SFA of these ratios was determined. Representative pictures of 100% Gal3^positive^ CSC (left), 100% Gal3^negative^ CSC (right), or 1:1 mixture (middle) are shown. *P*<0.05 (*); *P*<0.01 (**) and *P*<0.001 (***)

LiM6-TR and DLD1-TR are derivatives of colon cancer cell lines LS174T and DLD1 which are TRAIL resistant and have high cell surface and total Gal3 expression compared to their parental cell lines by flow cytometry, Western analysis, and confocal microscopy.^10^ Analogous to the flow cytometric analyses in Figure 1, we compared LiM6 to LiM6-TR and DLD1 to DLD1-TR, with respect to CSC (i.e., positivity for CD24, CD44, CD166, and EpCAM) and Gal3 positivity. Illustrations in Venn diagrams (Figure 3) show that Gal3 expression (blue plus red) is 3.0-fold higher (68 *versus* 23%) in the TRAIL-resistant LiM6 derivative and 7.2-fold higher (72 *versus* 10%) in the TRAIL-resistant DLD-1 derivative. LiM6-CSC and DLD1-CSC contain both Gal3^positive^ (in red) and Gal3^negative^ (in green) subsets (Figures 3a and b, left Venn diagrams).

Conversely CSC-subsets of LiM6-TR and DLD1-TR were all positive for Gal3 expression (Figures 3a and b, right Venn diagrams). We also analyzed the respective unsorted cell line pairs in comparative sphere-formation assays. Here enrichment for TRAIL-resistance is not only associated with Gal3 expression, but is also significantly associated with sphere number and sphere size, again strongly suggesting a role for Gal3 in identifying cancer cell stemness (Figures 3a and b, right bar graphs). Not all Gal3^positive^ cells, however, were also CSC. From 8.1 to 80.1% of Gal3^positive^ cells in various cell lines were also CSC, while for Gal3^negative^ cells, only 5.1 to 44.7% were CSC (Supplementary Fig. S1B). This suggests that Gal3 alone is not sufficient for CSC identification, but better defines CSC when combined with other markers.

To exclude artifactual effects and study the cellular hierarchy, we conducted a lineage-tracking experiment.^3^ For DLD1-TR cells, Gal3^positive^ CSC were transduced with a mCherry lentiviral reporter construct and Gal3^negative^ CSC with an eGFP lentiviral reporter construct (Figure 3c). After obtaining single cell solutions, both subsets were admixed at different ratios and subsequently examined in SFA experiments. The majority of spheres derived from Gal3^positive^ CSC (Figure 3d, bar graphs) and also formed larger spheres (Figure 3d, pictures), particularly when 50% or more Gal3^positive^ CSC were seeded.

### Effect of silencing of Gal3 on stemness behavior

**Figure 4:**
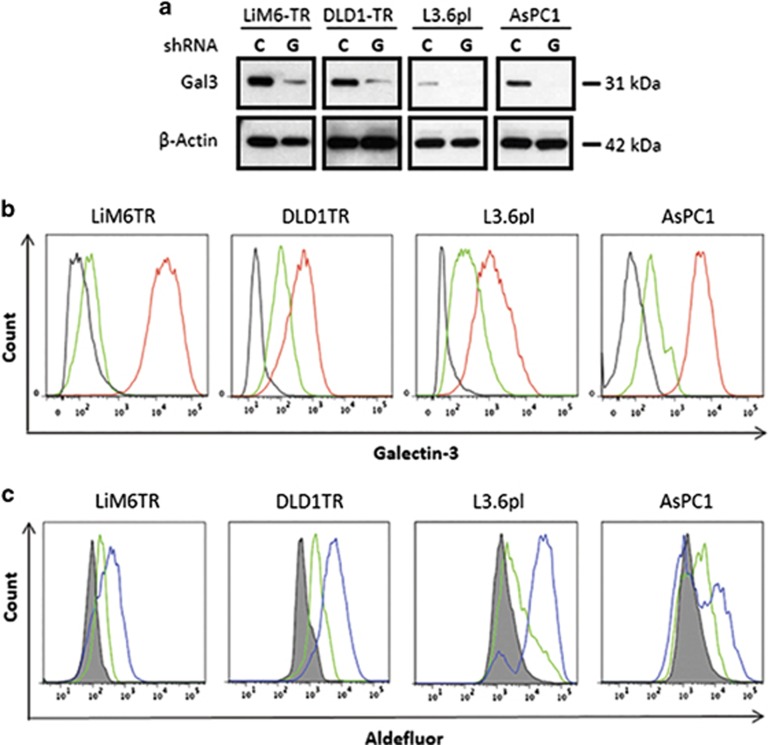
Silencing of Gal3 shifts CSC subset to a Gal3^negative^ CSC subset. (**a**) Western blot analysis for comparison of total Gal3 in LiM6TR or DLD1TR colon cancer cells and L3.6pl or AsPC1 pancreatic cancer cells after infection with lentiviral particles for control-shRNA (**c**) or Gal3-shRNA (G). Each pair of control and Gal3 knockdown cells were run side by side on the same gel. Comparison of Gal3 expression between cell lines has been previously published^10^ (**b**) Flow cytometric analysis of colon cancer and pancreatic cancer cells after infection with lentiviral particles for control-shRNA (red trace) or Gal3-shRNA (green trace). Black trace is background staining. (**c**) ALDH activity was analyzed by flow cytometry. ALDEFLUOR activity in colon cancer and pancreatic cancer cells after infection with lentiviral particles for control-shRNA (blue trace) or Gal3-shRNA (green trace). Gray profile is background staining

In order to more directly determine whether Gal 3 is a stem cell marker or is responsible for stem cell characteristics in CSC-subsets, we transduced cells with lentiviral particles harboring different Gal3-shRNAs (indicated as G-SH), which specifically depleted Gal3 protein. We chose LiM6-TR and DLD1-TR as colon cancer cell models, since both derivatives show relative enrichment of Gal3 and CSC subsets. Derivatives in which Gal3 was knocked down were termed LiM6TR-G-SH and DLD1TR-G-SH (colon cancer cells) and ASPC1-G-SH and L3.6PL-G-SH (pancreatic cell lines). Cells transduced with non-targeting control short hairpin-ribonucleic acid (sh-RNA) were designated C-SH for respective cell lines. Western blot analysis (Figure 4a) and flow cytometry (Figure 4b) confirmed depletion of total and cell surface Gal3.

In order to determine whether Gal3-depleted CSC is functionally different, we carried out ALDH sorting (Figure 4c). ALDH^positive^ populations were reduced in the Gal3-depleted LiM6TR-G-SH cells when compared to the control LiM6TR-C-SH cells (17.8 *versus* 69.1%). A similar pattern was observed in pancreatic cancer cells derived from L3.6pl (18.5% for G-SH *versus* 60.9% for C-SH). However, smaller differences were observed for DLD1TR derivatives (39.0 *versus* 47.8%) and AsPC1 derivatives (31.9 *versus* 33.5%).

**Figure 5:**
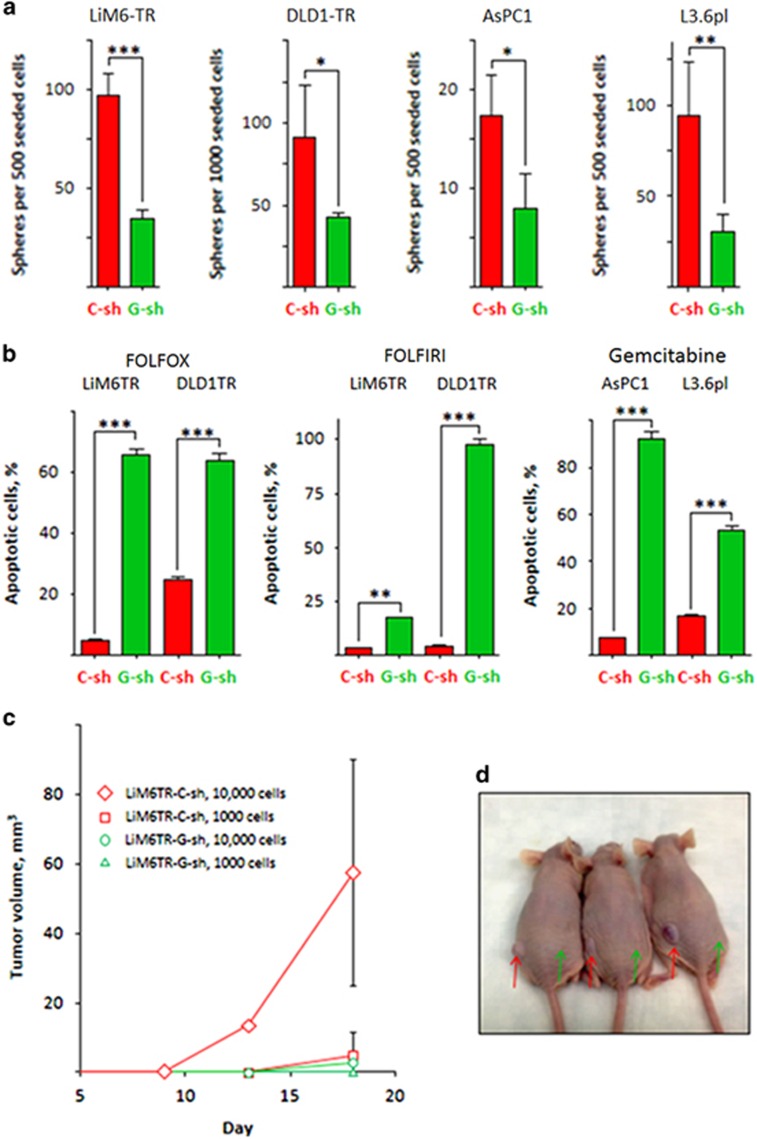
Effect of silencing of Gal3 on stemness behavior. (**a**) Knock-out of Gal3 (G-SH, in green) resulted in markedly decreased SFA compared to control cells (C-SH) in all cell lines. (**b**) Drug-resistance was significantly reduced in Gal3-depleted cells (G-SH, in green) as evaluated by APO-BRDU assays with conventional chemotherapy regimens (FOLFOX and FOLFIRI in colorectal cancer, left panels; gemcitabine in pancreatic cancer, right panel). (**c**) Tumor growth of Gal3^positive^ CSC (red) and Gal3^negative^ CSC LiM6TR cells (green) in subcutaneous model is shown. Gal3^positive^ CSC grow more rapidly with inoculation of 1 × 10^4^ (upper curve) and 1 × 10^3^ (lower curve) cells. (**d**) Representative picture of mice inoculated with 1 × 10^4^ cells derived from LiM6TR. Gal3^positive^ (red arrow), Gal3^negative^ (green arrow). All experiments were performed in triplicate. *P*<0.05 (*), *P*<0.01 (**), and *P*<0.001 (***)

In another functional assay, SFA was markedly reduced in all cell lines examined upon Gal3-depletion (G-SH, in green) compared to their respective controls (C-SH, in red) (Figure 5a, Supplementary figure 2).

Drug resistance is considered a characteristic of tumor-initiating stem cells.^20^ Gal3 has been associated with drug-resistance.^21, 22^ We therefore subjected Gal3-depleted colon cancer CSC and pancreatic cancer CSC to clinically relevant chemotherapeutic regimens, FOLFOX (5-fluorouracil, oxaliplatin, leucovorin) and FOLFIRI (5-fluorouracil, irinotecan. leucovorin) for colorectal cancer and gemcitabine for pancreatic cancer. All treatments consistently produced a high number of apoptotic cells in Gal3-depleted cells (in green), whereas Gal3^positive^ control cells (in red) were largely resistant to the applied drugs (Figure 5b).

The gold standard for CSC quantification is determination of tumorigenicity *in vivo* after limiting dilution.^23^ We xenografted LiM6TR-C-SH or LiM6TR-G-SH CSC subcutaneously into age-matched female athymic nu/nu mice in dilutions ranging from 10^3^ to 10^6^ cells. Injection of high cell numbers produced tumors for all cell lines. With injection of low cell numbers, LiM6TR-C-SH CSC tumors grew significantly faster and arose earlier compared to LiM6TR-G-SH CSC (Figure 5c). LiM6TR-C-SH CSC-derived xenografts were also larger and weighed more at the time of sacrifice (data not shown) compared to those derived from cells in which Gal3 was depleted from CSC. Mice inoculated with 10 000 cells each of Gal3^positive^ and Gal3^negative^ LiM6TR in different flanks developed tumors only from the Gal3^positive^ LiM6TR control cells(Figure 5d).

## Discussion

Recurrence of colorectal cancer after surgery and chemotherapy may be explained in part by the presence of chemoresistant CSC.^24^ Similarly, patients with pancreatic ductal adenocarcinoma also have a poor prognosis, which can be at least partially attributed to the observation that the standard chemotherapeutic agent gemcitabine is not capable of eliminating CSC and might, in fact, enhance the relative number of CSC.^25^ Hence, significant efforts are currently being undertaken to identify and eliminate CSC populations. However, the definitive identification of clinically relevant cancer subgroups that ultimately encumber patient survival through drug-resistance and metastatic spread remains a challenge, in part because commonly used stem cell markers (e.g. CD133, CD44, and CD166) may not accurately reflect these biological characteristics.^26^ Some markers may not be expressed at the protein level, for example, LGR5.^27^

Here, we present evidence that cell surface Gal3, while not exclusively limited to CSC, can better define such CSC subsets in colon and pancreatic cancers. Flow cytometric analysis of commonly used CSC markers (CD24, CD44, CD166, and EpCAM) revealed two subgroups of CSC distinguishable by differences in cell surface Gal3 expression. Additional investigation of functional CSC properties demonstrated that Gal3 expression on CSC was associated with increased SFA, ALDH activity, and enhanced tumorigenesis. Notably, we found that Gal3^positive^ CSC were not only resistant to death receptor-mediated apoptosis (TRAIL), but also to conventional chemotherapy regimens, such as FOLFIRI and FOLFOX for colorectal cancer or gemcitabine for pancreatic cancer. Gal3 expression alone was not sufficient for CSC identification, but better defined CSCs when combined with other canonical markers. Depletion of Gal3 led to a re-sensitization to TRAIL in cells with stably acquired TRAIL-resistance (LiM6-TR and DLD1-TR)^10^ and increased sensitivity to conventional chemotherapeutic regimens. Conversely, acquired drug-resistance was associated with increased cell surface Gal3-expression and an enlarged CSC pool within the bulk tumor. This is in keeping with the many biological functions previously reported for extracellular and intracellular Gal3.^28^ Substantial evidence indicates that Gal3 contributes to neoplastic transformation and tumor survival (including resistance to apoptosis), and other events associated with metastasis^28^ It is not known whether Gal3 expression is associated with all of the same functions in CSC as in non-stem cells, but several of the functions previously reported to be associated with Gal3 were enhanced in Gal3-positive CSCs. We have previously reported that Gal3 mediates Wnt signaling in unsorted colon cancer cells.^29^ This is also the case with spheres derived from colon cancer CSCs (Supplementary figure 3).

Our study demonstrates that cell surface Gal3 expression on colon and pancreas derived CSC are associated with an enhanced CSC phenotype. CSC appear to be heterogeneous in their expression of functional markers, and identification of CSC, including circulating tumor cells^30^ associated with enhanced tumorigenicity, metastatic potential and drug resistance, may provide new therapeutic strategies for targeting clinically relevant CSC.

## Materials and Methods

### Human colon and pancreatic cancer cell lines

DLD1 colon cancer cells and AsPC1 pancreatic cancer cells were obtained from the American Type Culture Collection (ATCC). AsPC1 is morphologically heterogenous in culture by phase contract microcopy. LS-LiM6 and L3.6pl are metastatic cell lines selected for increased liver metastasizing ability by serial *in vivo* passaging following orthotropic implantation in athymic nude mice. LS-LiM6 colon cancer cells were derived from human colon cancer cell line LS174T^31^ and L3.6pl pancreatic cancer cells were derived from the COLO357 pancreatic cancer cell line.^11^ Cell lines were validated by STR DNA fingerprinting using the AmpF_STR Identifier kit according to the manufacturer's instructions (Applied Biosystems, Foster City, CA, USA, cat 4322288). The STR profiles were compared to known ATCC fingerprints (ATCC.org), and to the Cell Line Integrated Molecular Authentication database (CLIMA) version 0.1.200808 (http://bioinformatics.istge.it/clima/). The STR profiles matched known DNA fingerprints of LS174T colon cancer cells. Cells were maintained at 37°C in a 5% CO_2_ atmosphere in Dulbecco's modified Eagle medium containing 10% heat-inactivated fetal bovine serum with penicillin and streptomycin.

### Generation of stable TRAIL-resistant cells

In order to secure stable TRAIL-resistant cells, parental LS-LiM6 or DLD1 cells were treated with 100 ng/ml human recombinant TRAIL (R&D Systems, Danvers, MA, #375-TEC) for 24 h. The apoptotic cells were then removed and the surviving cells were propagated in the presence of 10 ng/ml TRAIL for a total of five cycles resulting in stable TRAIL-resistant cell lines LiM6-TR and DLD1-TR. The sensitivity of TRAIL-resistant cell lines to TRAIL was periodically examined by PI (Sigma, St. Louis, MO) staining and flow cytometry, and never exceeded 5% cell death as described before.^10^

### Flow cytometry analysis and sorting

To identify and isolate CSC, parental cells were collected in Versene solution (Invitrogen, Carlsbad, CA, USA, cat#15040), washed and blocked with flow cytometry buffer (Santa Cruz Biotechnology, Dallas, TX, USA), labeled with the following primary conjugated antibodies and their matched isotypes: anti-CD24-PE-Cy7(561646), anti-EpCAM-FITC (BD Bioscience, San Jose, CA, USA, 347197) anti-CD44-APC-eFluor 780(47044182), anti-CD166-PerCPeFluor 710(46166842), anti Gal3-PE(12-5301-83) (all from - eBioscience, San Diego, CA, USA). LIVE/DEAD Fixable Dead Cell Stain (Invitrogen) was used for exclusion of dead cells, and isotype-matched control antibodies were used to set gates for marker positivity in viable single cells. Single cells were identified based on gating hierarchy using a plot of forward scatter area (FSC-A) *versus* side scatter area (SSC-A) followed by a doublet discrimination gate on a SSC-A *versus* SSC-H plot. CSC were acquired by flow cytometry using a LSRFortessa (BD Biosciences), Coulter Epics XL (Beckman Coulter, Brea, CA, USA), or Gallios (Beckman Coulter) and analyzed with FlowJo 8.8.6 software (Treestar, Ashland, OR, USA). The CSC subpopulations were isolated by sorting on an Influx Sorter using the sort ware 1.0 software (BD Biosciences).

### Sphere culture and sphere-formation assays

Parental cells were trypsinized, washed with PBS, and seeded in a clonal density of (5 × 10^3^/mL) in CSC medium consisting of DMEM supplemented with L-Glutamine, B27, rhEGF (10 ng/ml)/rhFGF (10 ng/ml), and Penicillin/Streptomycin in ultra- low-attachment plates (Corning, Corning, NY, USA). Cell medium was changed every 3–4 days and spheres were trypsinized and reseeded for expansion. All CSC markers and surface Gal3 expression were maintained without significant change up to seven generations. All CSC markers and surface Gal3 expression were maintained without significant change up to the seventh generation. For quantification of SFA, single cell suspensions were seeded into ultra-low-attachment 96-well plates (500–1000 cells/100 *μ*l) in CSC plus 1% methylcellulose. Hundred microliters of the same medium was added after 3 days and exchanged after another 3-4 days. Spheres were usually counted after 10 days. Only spheres >75 *μ*m in diameter were included, if not otherwise stated.

### Lineage tracking

The Gal3^positive^ CSC subset derived from DLD1-TR was genetically labeled using lentiviral particles expressing mCherry (GeneCopoeia) and the Gal3^negative^ with GFP (Santa Cruz Biotech) and further sorted to ensure uniform labeling. Gal3^positive^ (mCherry) and Gal3^negative^ (GFP) cells were reconstituted at the desired ratios, and then cultured in a low cell density allowing for clonal expression. After 2 weeks of culture, the remaining cells were examined for mCherry/GFP expression and counted by fluorescence microscopy.

### ALDH assay

Spheres were dissociated into single-cell suspension and allowed to recover for 24 h in CSC media in low-attachment 6-well plates. ALDH activity was monitored using the ALDEFLUOR kit (STEMCELL Technologies, San Diego, CA, USA) following the manufacturer's instruction. The ALDH activity was analyzed by flow cytometry using the LSRFortessa and quantified by FlowJo 8.8.6 Multicycle cell software. Single cells were identified based on gating hierarchy using a plot of FSC-A *versus* SSC-A followed by a doublet discrimination gate on a SSC-A *versus* SSC-H plot.

### Western blots

Protein sample preparation and western blot analysis were carried out as described previously.^8^ Briefly, whole cell extracts were lysed in SDS lysis buffer and were resolved on SDS-PAGE gels, and then transferred to a nitrocellulose membrane. Proteins were detected using rat monoclonal antibody against Gal3 (1:500, ascites fluid of TIB-166, ATCC# M3/38.1.2.8 HL.2) and beta-actin (1:5000 AC-15, Sigma,A5316). Protein bands were visualized using an enhanced chemiluminescence detection kit (ECL Advance, GE Healthcare, Marlborough, MA, USA).

### Apoptosis and cell cycle assays

Spheres were dissociated into single-cell suspension and 1 × 10^6^ cells were seeded in 2 ml of CSC media in 6-well low-attachment plates and allowed to recover for 24 h. The spheres from all cell lines were treated with 100 ng/ml TRAIL, while spheres originated from the colon cell lines were exposed to a combination of either 50 *μ*M 5FU (F6627), 20 mM Leucoverin (F7878), and 1.25 *μ*M Oxaliplatinum (O9512) (FOLFOX) or 50 *μ*M 5FU, 20 mM Leucoverin, and 50 mg/ml Irinotecan (I1406), (FOLFIRI) for 24 h. Spheres from the pancreatic cell lines were treated with 20 *μ*g/ml of Gemcitabine for 48 h. (All inhibitors were purchased from Sigma Aldrich, St. Louis, MO, USA.) Apoptosis was assessed by the Apo Alert BrdU assay (BD Bioscience) according to the manufacturer's instruction and quantified by flow cytometry, on a BCI Gallios (Coulter Counter, Brea, CA, USA) and analyzed with the FlowJo software. Cell cycle distribution was analyzed by Multicycle software.

### Proliferation assay

Spheres were dissociated to single-cell suspension and 1000 cells were seeded in CSC medium in low- attachment 96-well plates. Cell growth was determined every 48 h for 5 days by the MTT (3-(4,5-Dimethyl-2-thiazolyl-2,5-diphenyl-2H-tetrazolium bromide) assay kit (Promega, Madison, WI, USA) according to the manufacturer's instructions. Colorimetric reading at 570 nm was performed using an MRX Revelation micro plate reader (Dynex, Chantilly, VA, USA). The results are expressed as the mean OD from triplicate determinations.

### Silencing of Gal3 expression

Cells were infected with the Gal3 sh-RNA (#sc-155994-V) and non-targeting control sh-RNA (#sc-108080) lentiviral particles (Santa Cruz Biotechnology) according to the manufacturer's protocol. After transduction, stable cell lines expressing the Gal3 (or control) sh-RNA were isolated by selection with puromycin. Protein expression by western blot analysis or cell surface expression by flow cytometry were verified following propagation for four passages.

### *In vivo* xenograft experiments

Mouse experiments were performed in accordance with the Institutional Animal Care and Use Committee (IACUC) at MD Anderson. Immunocompromised age-matched male 5- to 6-weeks-old, athymic, nu/nu, Balb/c mice were purchased from an institutional breeder colony and kept at 24 °C in sterile conditions with water and food ad libitum. Different dilutions of LiM6TR-C-SH- or LiM6TR-G-SH-CSC-cells were subcutaneously injected in 50 *μ*l PBS in the left and right flank respectively via a 27G-needle. Tumor growth was controlled and measured regularly and mice were checked for their vital status and weight. Tumor volume was calculated by the following formula: mm^3^=(width × width × length)/2 [mm]. All mice were sacrificed when their tumor volumes reached high tumor burdens according to the IACUC protocol or at the end of the experiment. Subsequently, tumor parts that were selected for further *in vitro* cell culture experiments were fixed in 4% paraformaldehyde for histological analyses.

### Statistical analyses

Results are expressed as the mean±standard error of the mean (S.E.M.). All statistical comparisons were made with a standard *t*-test, using biostatistics software from GraphPad Prism (La Jolla, CA). The criteria for significance were *P*<0.05 (*), *P*<0.01 (**), and *P*<0.001 (***) for all comparisons. All experiments were performed at least in triplicate.

## Abbreviations

CSC: cancer stem cells
Gal3: galectin-3
TRAIL: tumor necrosis factor-related apoptosis-inducing ligand
SFA: sphere forming ability
FOLFOX: 5-fluorouracil, oxaliplatin, leucovorin
FOLFIRI: 5-fluorouracil, irinotecan, leucovorin
sh-RNA: short hairpin-ribonucleic acid
