# Supplementary figures and images for: Cell surface galectin-3 defines a subset of chemoresistant gastrointestinal tumor-initiating cancer cells with heightened stem cell characteristics
Source: Cell Death Dis. 2016 Aug 11;7(8):e2337–. doi: 10.1038/cddis.2016.239 (PMC5108324; doi:10.1038/cddis.2016.239)

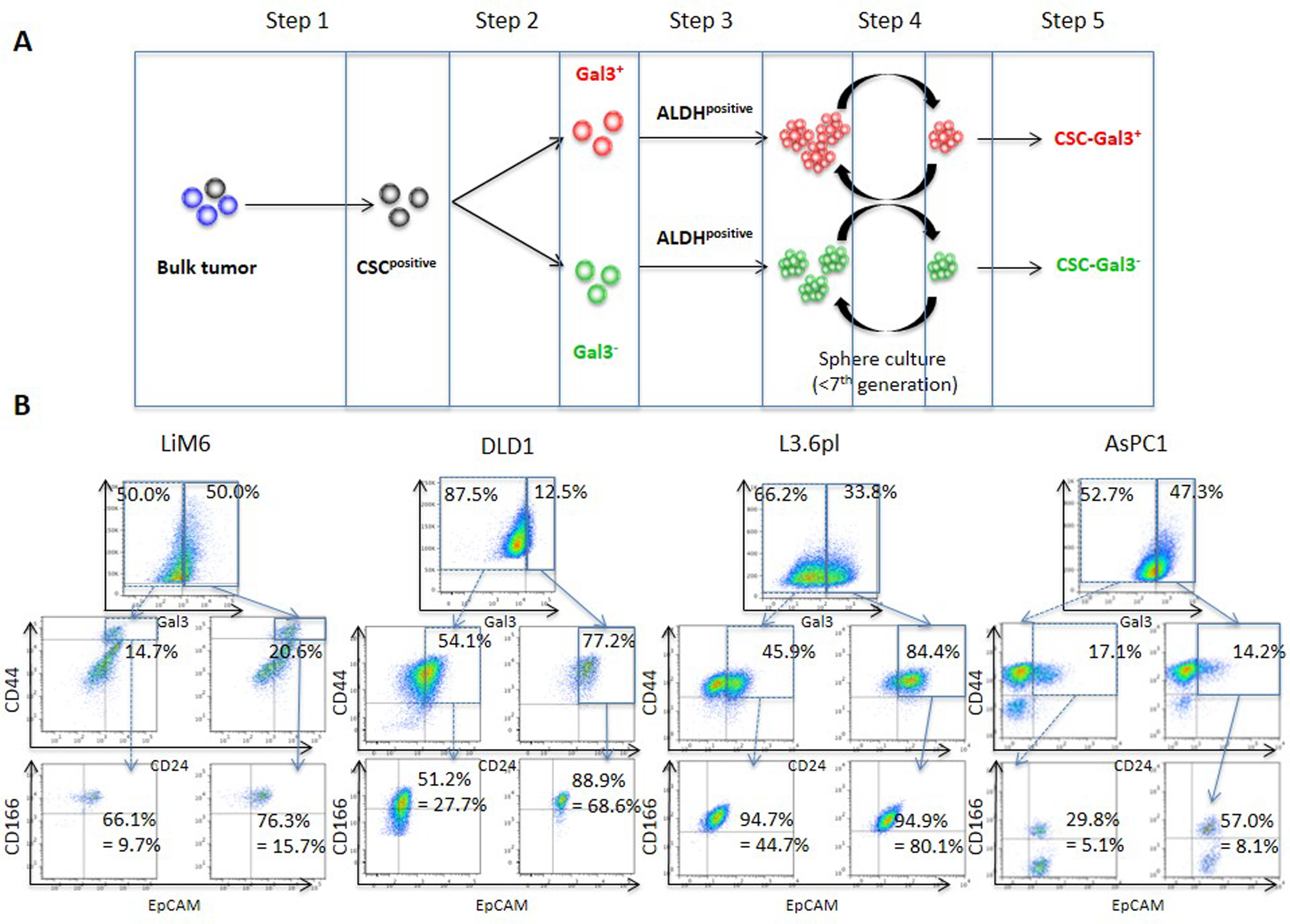

Supplement: Supplementary Figure 1 [file cddis2016239x1.tif]

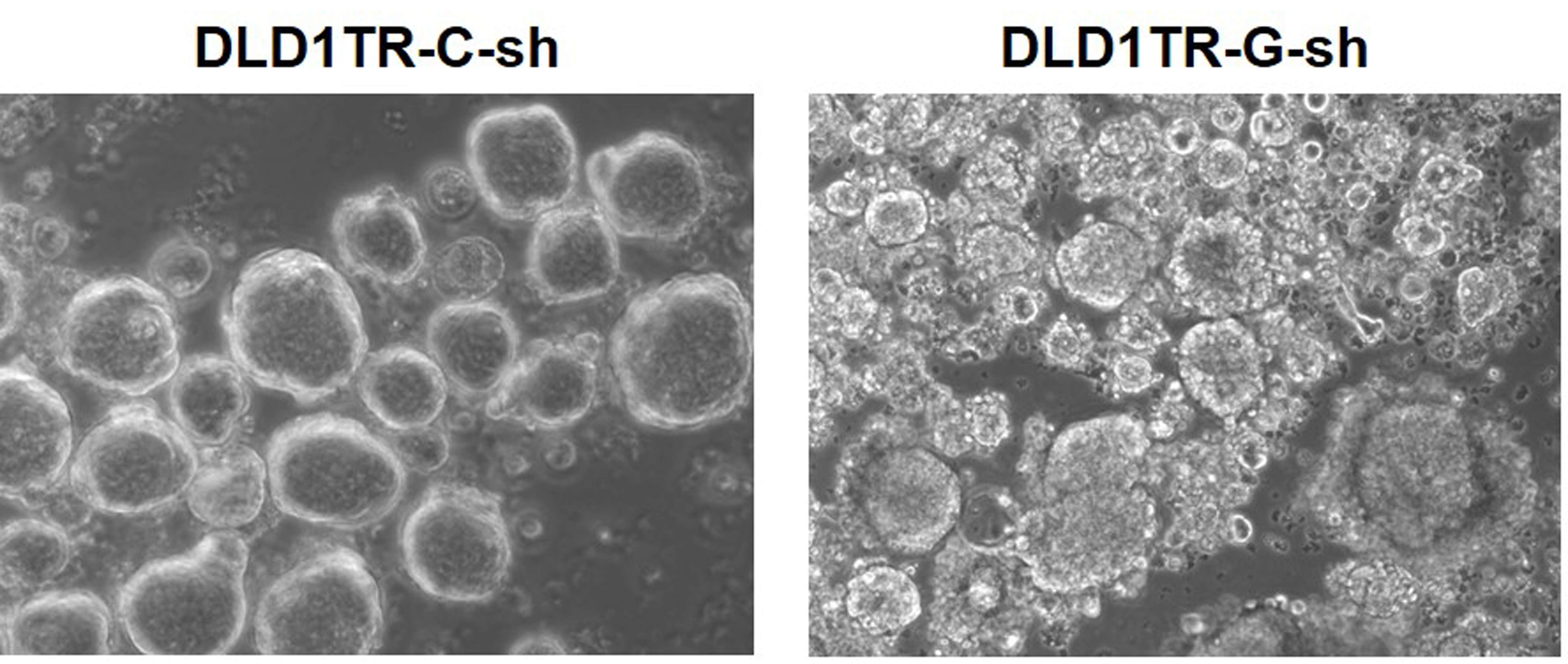

Supplement: Supplementary Figure 2 [file cddis2016239x3.tif]

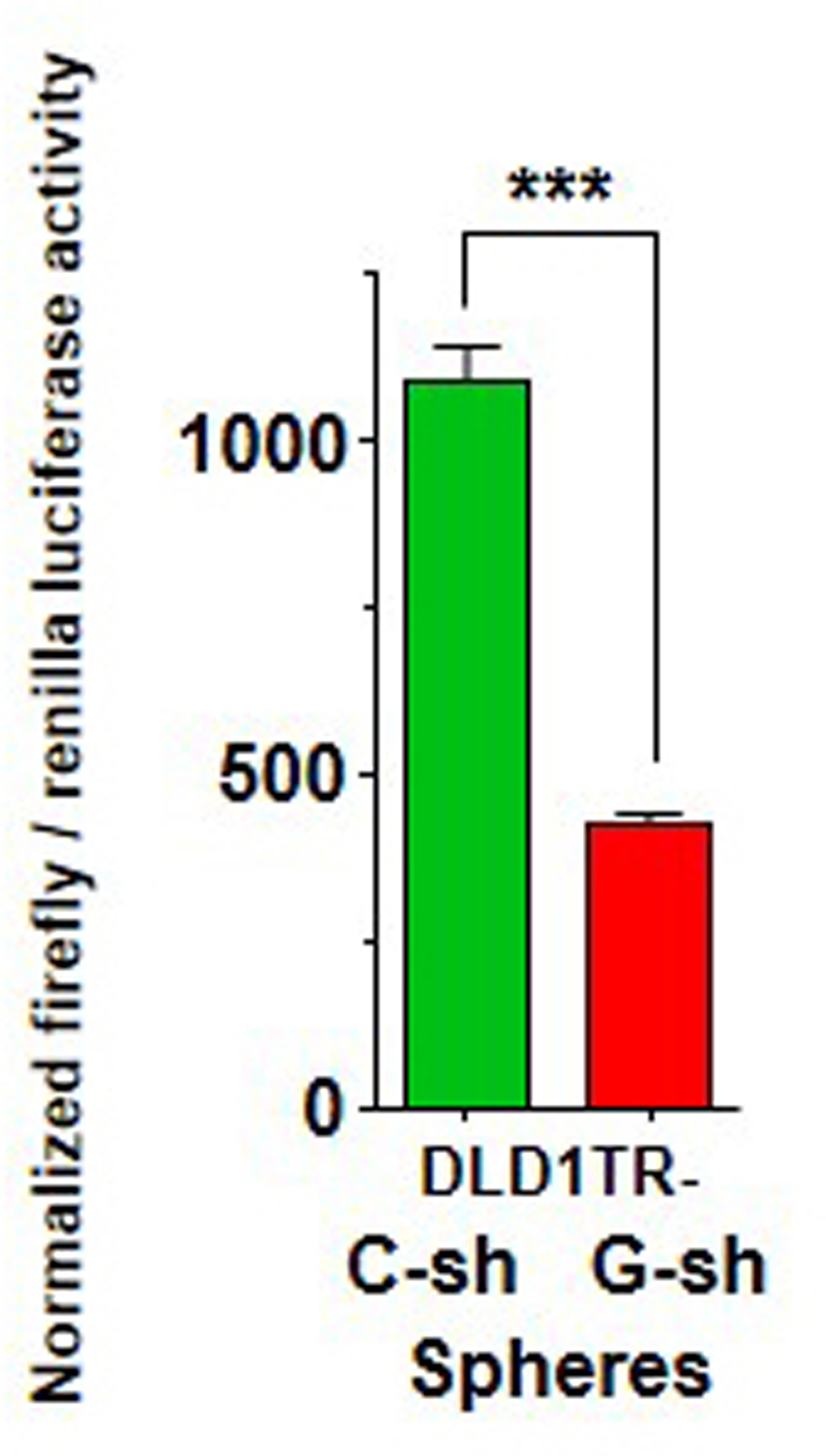

Supplement: Supplementary Figure 3 [file cddis2016239x5.tif]
