# Supplementary material for: Cell surface galectin-3 defines a subset of chemoresistant gastrointestinal tumor-initiating cancer cells with heightened stem cell characteristics
Source: Cell Death Dis. 2016 Aug 11;7(8):e2337–. doi: 10.1038/cddis.2016.239 (PMC5108324; doi:10.1038/cddis.2016.239)
Supplement: Supplementary Figure 1 Legend [file cddis2016239x2.doc]

**Figure S1.**

(A) Graphical presentation of the workflow. (Step 1): bulk tumor cells were sorted for CD24^+^/CD44^+^/CD166^+^/EpCAM+ (CSC). (Step 2): CSC were separated into Gal3Positive (red) and Gal3^Negative^ (green) subsets. (Step 3): both subsets were further sorted for ALDHpositive cells and then maintained as spheres for up to 7 generations. (Step 4): Last, these spheres were investigated in further assays and marker expression verified periodically (Step 5). (B) Flow cytometry results of Figure 1B and C were reversely analyzed. First, cells were divided into Gal3Positive or Gal3Negative cells (upper panels). Then, both subsets were analyzed on their CD24^+^/CD44^+^ subsets (middle panels) and the latter then evaluated for their CD166^+^/EpCAM^+^ subsets (lower panel).
