# Supplementary material for: Cell surface galectin-3 defines a subset of chemoresistant gastrointestinal tumor-initiating cancer cells with heightened stem cell characteristics
Source: Cell Death Dis. 2016 Aug 11;7(8):e2337–. doi: 10.1038/cddis.2016.239 (PMC5108324; doi:10.1038/cddis.2016.239)
Supplement: Supplementary Figure 2 Legend [file cddis2016239x4.docx]

**Figure S2.**

**Loss of Gal3 impedes sphere formation capacity.**

DLD1TR cells were transduced with non- targeting control sh-RNA (DLD1TR-C-sh, left) and cells in which Gal3 was knocked down by transduction with lentiviral particles harboring different Gal3-shRNAs (DLD1TR-G-sh, right) were examined for sphere-forming ability.
