# Supplementary material for: Cell surface galectin-3 defines a subset of chemoresistant gastrointestinal tumor-initiating cancer cells with heightened stem cell characteristics
Source: Cell Death Dis. 2016 Aug 11;7(8):e2337–. doi: 10.1038/cddis.2016.239 (PMC5108324; doi:10.1038/cddis.2016.239)
Supplement: Supplementary Figure 3 Legend [file cddis2016239x6.docx]

**Figure S3.**

**Loss of Gal3 results in reduced Wnt-Signaling activity.**

For transient Wnt reporter assays using the Super TOP/FOP system, 5.0x10^4^ spheroid cells were transduced with 500 ng inducible firefly luciferase expressing either SuperTOP or SuperFOP vector (AddGene Plasmids #12456 and #12457) and cotransfected with constitutively Renilla luciferase expressing normalization control vector pRL-TK (Promega) at a ratio of 50:1. Spheres were then cultured for 24 hours, lysed with passive lysis buffer, and firefly and Renilla luciferase activity was evaluated with the Dual Luciferase Reporter Assay System (Promega) according to the manufacturer's instructions. All experiments were performed in triplicate.
